# Identification, Computational Examination, Critical Assessment and Future Considerations of Distance Variables to Assess Collective Tactical Behaviour in Team Invasion Sports by Positional Data: A Systematic Review

**DOI:** 10.3390/ijerph17061952

**Published:** 2020-03-17

**Authors:** Markel Rico-González, José Pino-Ortega, Fabio Y. Nakamura, Felipe Arruda Moura, Asier Los Arcos

**Affiliations:** 1 Department of Physical Education and Sport, University of the Basque Country, UPV-EHU. Lasarte 71, 01007 Vitoria-Gasteiz, Spain; markeluniv@gmail.com; 2 Faculty of Sports Sciences, University of Murcia, 30720 San Javier, Spain; pepepinoortega@gmail.com; 3 Associate Graduate Programme in Physical Education UPE/UFPB, João Pessoa, 58051-900 Paraíba, Brazil; fabioy_nakamura@yahoo.com.br; 4 Laboratory of Applied Biomechanics, Sports Sciences Department, State University of Londrina, Av. Gil de Abreu e Souza, 2335, Unidade 1121, Esperança, Londrina 86058-100, Brazil; felipemoura@uel.br

**Keywords:** team behaviour, tactic, dyad, entropy, relative phase

## Abstract

The aim of the study was the identification, computational examination, critical assessment and future considerations of *distance* variables to assess collective tactical behaviour in team invasion sports by positional data. A total of 3973 documents were initially retrieved. Finally, 72 articles met the inclusion criteria, but only 26 suggested original tactical variables based on the *distance* variables. The *distance* variables can be classified into player–player, player–space, player–ball, and Geometrical Centre (GC)–GC /player/space/goal. In addition, several nonlinear techniques have been used to analyse the synchronisation and predictability of the *distance* variables in team invasion sports. Player–opponent *distance* is of special interest in those sports in which man-marking is commonly used, and in the micro-structure close to scoring situations in all sports. In addition, player–player distances are used to measure the length and the width of the team and player–GC *distance* to assess the dispersion of the team. Player–space distances have been measured to assess the *distance* of the player/team-line to relevant areas of the playing space. Several techniques have been applied to analyse the synchronisation (i.e., Hilbert transformation and cluster analyses) and the complexity and regularity or predictability (i.e., approximate entropies, sample entropy, cross-sample entropy and average mutual information) of the *distance* variables in team invasion sports, revealing the lack of consensus. Although the *distance* variables may be interesting tactical variables when considered in isolation, it would be enriching to analyse the relationship among these variables.

## 1. Introduction

Although all players constantly interact with one another during team invasion sports matches and tasks, the nature [1] of these interactions differs considerably, according to the location of the ball [2,3], the location of players with respect to the goal [4,5], and the team in possession of the ball [6,7]. For this reason, the decomposition of the team into micro-structures (or sub-systems [1]) has been suggested in order to assess team behaviour. This decomposition means a reductionist approach [1] of the social system (i.e., a collective duel) [8,9,10], but allows analysis of relevant and special interactions among several players. Tactical behaviours can be assessed at the individual, dyadic, sub-group (e.g., sectorial) and/or team level [9,11,12,13]. One of the most analysed micro-structures in team invasion sports has been the dyad [11,12,13].

Based on studies of racket games [14], the assessment of the interaction of two players (i.e., *distance* variables) has been suggested through the measurement of the *distance* between both players (i.e., player –player, and player–opponent) [4,5,15,16,17,18,19,20]. In addition, several studies have considered the *distance* of the players in a particular zone of the pitch; that is, they have measured several player–space distances [5,21,22]. After more than twenty years, many types of *distance* variables have been used to assess team behaviour in team invasion sports [4,5,7,16,17,18,20,21,23,24,25,26,27]. However, it would be interesting to classify and analyse these *distance* variables in order to assess their practical application in team invasion sports training and matches.

The inherent complexity of team invasion sports [28] suggests the use of nonlinear tools to analyse the synchronisation and predictability of the *distance* variables [12]. These types of analysis are essential to understanding the dynamics and the performance in team invasion sports [28]. Based on previous studies that examine bimanual coordination [29] and periodic movements with one limb while watching each other [14], Palut and Zanone [14] computed the relative phase to assess epoch synchronisation in tennis. This was suggested as a collective variable to capture the modes of movement that two oscillators demonstrate during games (i.e., in-phase and anti-phase [14]). The relative phase has been widely used to assess the synchronisation between several types of oscillators [2,15,30,31]. On the other hand, several measures of entropy [28] have been proposed to assess the results, the complexity, the regularity or the predictability of the time series of a system [17,19,20,32] of nonlinear dynamical systems as an example (i.e., team invasion sports [19]). While a decrease in entropy reflects a decrease in unpredictability, a high entropy means that the minimum information necessary to describe the system has increased with system variability and its behaviour is more unpredictable [28]. Thus, it is necessary to review the origin, application [33], and different mathematical concepts and computations applied [19,30] to identify the differences in the measurement of the relative phase and entropy in the *distance* variables.

The aim of the present study was the identification, computational examination, critical assessment and future considerations of *distance* variables to assess collective tactical behaviour in team invasion sports by positional data.

## 2. Materials and Methods

### 2.1. Search Strategy

This systematic review was reported in accordance with the Preferred Reporting Items for Systematic Reviews and Meta Analyses (PRISMA) guidelines [34]. The protocol was not registered prior to initiation of the project and did not require Institutional Review Board approval. A systematic search of four databases was performed by the authors (MR, ALA, JPO) to identify articles published before 13 November of 2018. The authors were not blinded to journal names or manuscript authors. The PICO [34] design was used to provide an explicit statement of question. The search was carried out using two filters where the database allowed this (journal article and title (TI)/abstract), except in WoS, which was searched throughout the text. In addition, in the last-mentioned database in the sports sciences branch was selected. The search was made using combinations of the following terms linked with the Boolean operators “AND” (inter-group Boolean operator) and “OR” (intra-group Boolean operator). Three main groups were created: (1) “Soccer”, “football”, “team sport*”, “basketball”, “rugby”, “handball”, “hockey”; (2) “GPS”, “global position system*”, “GNSS”, “Global navigation satellite system*”, “UWB”, “ultra wide band”, “local position”, “LPP”, “LPS”, “LPS”, “EPTS”, “electronic performance and tracking systems*”, “video”, “video tracking”, “tracking system*”, “electronic*”, “satellite system*”, “GIS”, “geographical information system*”; and (3) “formation*”, “tactic*”, “behaviour*”, “performance*”, “position*”, “spatiotemporal”, “spatio-temporal”, “synchronization*”, “coordination*”, “pattern*”, “synerg*”, “Voronoi”, “Delaunay”, “decision-making”, “decision making”.

### 2.2. Screening Strategy and Study Selection

When the referred authors had completed the search, they compared their results to ensure that the same number of articles had been found. Then, one of the authors (MR) downloaded the main data from the articles (title, authors, date, and database) to an Excel spreadsheet (Microsoft Excel, Microsoft, Redmond, USA) and removed the duplicate records. Subsequently, the same authors screened the remaining records to verify the inclusion/exclusion criteria, using a hierarchical approach in two phases: Phase 1, titles and abstracts were screened and excluded by two authors (MR, ALA) against criteria 1–5 where possible; Phase 2, full texts of the remaining papers were then accessed and screened against inclusion criteria 1–5 by the same authors (MR, ALA). The papers that were included after these phases and met the 6th inclusion criteria were included in the systematic review. The inclusion criteria were: (1) Team sports in which the use of the mobile (e.g., ball, puck) is simultaneous (e.g., soccer, hockey); (2) The main objective of the study is to assess tactical performance or dimension in team players; (3) Studies that include a tactical variable regarding the position of the players using Electronic Performance and Tracking Systems (EPTS); (4) Studies that aim to measure a tactical variable; (5) Studies that aim to analyse the position of more than one player, whether they are rivals or not; (6) Studies that measured the *distance* variables or modified this variable, and provided their computational criteria. The studies included in the review met all inclusion criteria. The quality of the included studies was individually assessed using a modified Downs and Black checklist by Sarmento et al. [35].

Any disagreements on the final inclusion/exclusion status were resolved through discussion in both the screening and excluding phases. Moreover, relevant articles not previously identified were also screened in an identical manner and the studies that complied with the inclusion/exclusion criteria were included and labelled as ‘not identified from search strategy’.

## 3. Results

**Figure 1:**
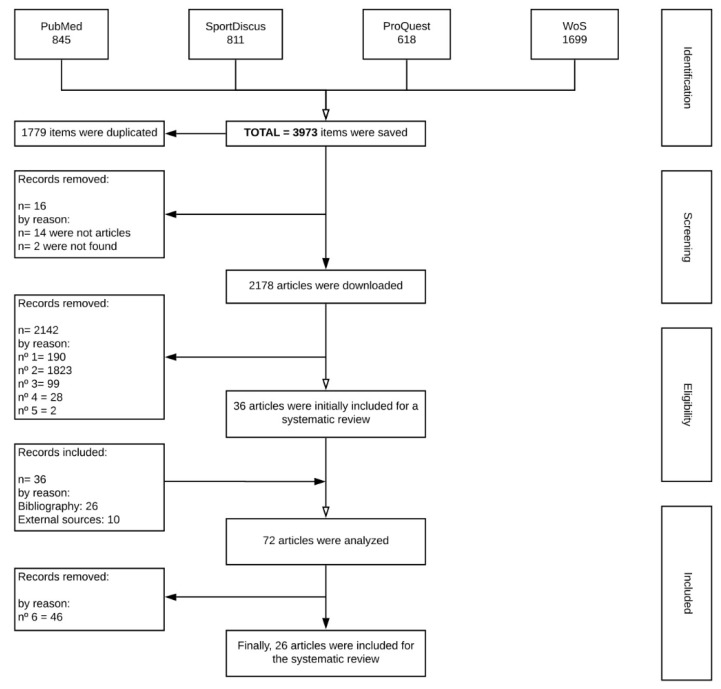
Flow diagram of the study.

A total of 3973 documents were initially retrieved, of which 1779 were duplicates. A total of 2178 articles were screened. Next, the titles and abstracts were verified against criteria 1–5 and studies were excluded where possible. The full texts and abstracts of the remaining articles were screened and the inclusion/exclusion criteria were applied, leading to the exclusion of 2142 articles. Therefore, 36 articles were initially included in this review having met the inclusion criteria 1–5. In addition, the authors found and added 36 articles that met inclusion criteria 1–5. Finally, 72 works were analysed and 26 articles were included in the systematic review after meeting the 6th inclusion criterion (Figure 1). Among them, eighteen were originals or showed modification of *distance* variables (Table 1 and Table 2) and twelve were originals or proposed modifications of non-linear techniques (Table 3).

## 4. Discussion

The aim of the study was the identification, computational examination, critical assessment and future considerations of *distance* variables to assess collective tactical behaviour in team invasion sports by positional data. According to the nature of the oscillators or points, the *distance* variables can be classified into player–player, player–space, player–ball, and geometrical centre (GC)–GC/player/space/goal. Player–opponent distances are of special interest in those team invasion sports in which man-marking is commonly used and in the micro-structure close to scoring situations in all team invasion sports. In addition, player–player distances are used to measure the length and the width of the team and player–GC distances to assess the dispersion of the team. The player–space distances have been measured to assess the *distance* of the player/team-line to relevant areas of the playing space. Several techniques have been applied to analyse the synchronisation (relative phase by the Hilbert transformation and the cluster analyses) and the complexity and regularity or predictability (various approximate entropies, sample entropy, cross-sample entropy and average mutual information (AMI)) of the distances in team invasion sports, revealing the lack of consensus among researchers.

The interaction between two players, assessed in *distance* (i.e., dyad [13,41]), is the most commonly analysed micro-structure in team invasion sports [2,5,7,16,17,18,21,24,26], although the same concept has been also used to assess the *distance* between different types of oscillators or points (i.e., points of union): GC–GC, GC–player, GC–space, GC–ball, player–space, player–ball [7,20,25,39,42]. In fact, the first proposed *distance* variables considered the *distance* between the player and the basket [41].

### 4.1. Player–Player

#### 4.1.1. Player–Opponent

The measurement of distances in team invasion sports was suggested by Araujo et al. [43]. Specifically, the author proposed the positional balance between attacker and defender in basketball [43]. Although Passos et al. [22] considered the attacker and defender as oscillators, they calculated the *distance* of each player from the try and lateral lines. Thus, Passos et al. [24] measured the *distance* between a defender and their opponent (i.e., player–opponent) for the first time. The authors compared the impact of both interpersonal *distance* and relative velocity on attacker–defender distances during an experimental task that was representative of a typical sub-phase of rugby union (i.e., 1 vs. 1 near the try line) [24]. Next, Bourbousson et al. [16] measured the *distance* between the attacker and the defender in fixed player–opponent distances (i.e., the players of the *distance* do not change during the analysis) in a basketball match and Silva et al. [20] calculated the distances separating each player from their nearest opponent, that is, no fixed player–opponent distance, in order to assess their uncertainty during soccer small-sided games (SSG) and conditioning games. Thus, player–opponent distances (i.e., individual duels) have been assessed during play considering the same two opponents (i.e., fixed *distance* variables continuously, and varying the opponents of the *distance* during play). Fixed player–opponent *distance* variables are of special interest in those sports in which man-marking is commonly used, such as basketball [16] and futsal [3], although it is also interesting to measure this micro-structure close to scoring situations in other team invasion sports such as soccer and rugby [5,24,44]. Non-fixed player–opponent *distance* variables could be more relevant in sports in which zonal marking is applied by the trainers.

Based on the defender–attacker *distance* variables, Silva et al. [20] proposed team separateness (TS), the sum of distances between each team player and the closest opponent (i.e., a collective computation) during small-sided and conditioned games (SSCG), because this could be a more interesting variable than the GC to analyse the pressure exerted by one team on another. The authors defined TS as a measure of the degree of free movement that each team has available [20]. Silva et al. [36] proposed a modification of the TS. They understood the TS as the average *distance* between all players and their closest opponent and this was interpreted as the average radius of action free of opponents [36]. Based on the *distance* between the opponents, Silva et al. [4] proposed the measurement of the distances separating the teams’ horizontal and vertical opposing line-forces in order to examine inter-team coordination. As the authors explained, they assessed these variables instead of the GC because the former did not capture the existence of eventual differences in the players’ interactive behaviours at specific team locations (e.g., wings and sectors) [4]. The idea in this study was to calculate two horizontal lines and two vertical lines per team in several SSGs [4]. Each team’s horizontal lines were calculated by averaging the longitudinal coordinate values of the two players furthest from, and nearest to, their own goal line, which corresponded to the forward and back lines, respectively. Similarly, the vertical line-forces of each team were computed by averaging the mean lateral coordinates of the players furthest to the left and to the right of the pitch, corresponding to the left and right lines, respectively [4]. Finally, Shafizadeh et al. [26] assessed the distances between the shooter and the goalkeeper, regarding this measure as a candidate action-relevant variable informing goalkeepers about co-adapted positioning needed for goal saving.

#### 4.1.2. Player–Teammate

Another player–player distance, the *distance* between two teammates, has been widely assessed in team invasion sports [16,17,18,37]. The first time that the *distance* between two teammates was assessed, Lames et al. [37] measured the *distance* between the maximum and minimum position of the players (i.e., non-fixed *distance* variables) of the same team (i.e., the range per team) in order to assess the occupation of the space in the direction of goal to goal. Soon after, several studies proposed the measurement of the range in both directions (i.e., length and width) [45,46]. Later, Bourbousson et al. [16] assessed the player–teammate *distance* variable, but considering the *distance* between fixed *distance* variables (i.e., two playing positions), that is, the *distance* between the same teammates during a basketball match. A further study took into consideration all possible player–teammate *distance* variables formed by the outfield players in order to asses intra-team relations (the absolute values (m) and variability in the *distance* between players) [31]. Moreover, Olthof et al. [18] proposed different non-fixed player–teammate *distance* variables that considered the goalkeeper. Specifically, they measured the *distance* that remained behind the defensive line, which was a measure of the *distance* between the goalkeeper and the last defender. Thus, player–opponent and player–teammate *distance* variables were analysed independently in order to observe the positional balance between both players [22], and then as a collective index considering all or several *distances* [16,17,18,37,47] in order to assess intra-team and inter-team distances.

### 4.2. Player–Space and Player–Ball

As mentioned above, Passos et al. [22] measured a *distance* variable in team invasion sports for the first time. Specifically, a player–space distance: the *distance* of the attacker and the defender from the try line (i.e., absolute *distance* of each player from the try line over time, calculating the *distance* along a straight line between the closest point of the try-line and each player) and *distance* of attacker and defender from both lateral lines (i.e., absolute *distance* of each player from the lateral lines) during a rugby training task. Besides presenting a 3-D analysis of interpersonal dynamics of attacker-defender distances, the authors also aimed to identify parameters to measure dynamical system properties in these *distance* variables [22]. Similarly, Vilar et al. [5] suggested measurement of the difference between the attacker’s and defender’s distances to the centre of the goal (i.e., relative *distance* to the goal) in order to analyse how players coordinate their actions to create/prevent opportunities to score goals in futsal matches. Moreover, they proposed the assessment of the defender´s angle to the goal and the attacker (i.e., inner product of the defender´s vector to the centre of the goal, and the defender´s vector to the attacker) [5]. Maybe further studies should consider and add the influence (i.e., distance) of the goalkeeper in this type of analysis. In the same line of thought, Esteves et al. [21] linked the *distance* between the ball carrier and their immediate defender (i.e., player coordinates) and the *distance* of the ball carrier to the basket (i.e., player and space coordinates) in order to assess the *distance* between these two points when a shot is attempted or when possession is lost during competition basketball games. Thus, player–space distances have been measured taking into account the player–opponent relative position and *distance* to the goal, basket, or end zone [5,21,22]. Regarding the player–ball distance, Yue et al. [7] measured this type of *distance* variable for the first time, with several studies considering the position of the ball in the analysis of the team behaviour variables [3].

### 4.3. GC–GC/Player/Space

Taking into account the relative positioning of each team, expressed in single *x* and *y* coordinates, Frencken and Lemmink [6] measured the *distance* between the GCs of the teams in order to assess the “pressure” between the teams. Later, several studies proposed other tactical variables [20] as the GC–GC could be an excessive reduction in the relation between both teams. Since the same GC location can be due to very different player positions, it is necessary to assess the location of the players with respect to the GC of the team (i.e., the dispersion). Together with the computation of the GC, Yue et al. [7] proposed the measurement of the instantaneous radius (also named stretch index or spread [7]) of each team, calculating the average *distance* between all players and the GC of the team at that moment. In a later study, Barttlet et al. [23] picked up Yue’s idea [7] but applied a new calculation formula. It summarised the distances of all players from the team GC (*x_c_*), and because the team GC is computed from the position (*x_i_*) of all players, then the stretch index incorporates all inter-player distances.

The radius was used to analyse the counterphase relation in which it was observed how the team with possession expanded against the contraction of the defending team [7]. In 2012, Sampaio and Maçãs [25] proposed the measurement of the absolute *distance* between the GC and each player to assess the coordination of each player and GC using the relative phase (this data processing technique will be discussed in the next section). In addition, these authors proposed the measurement of the maximal and minimal *distance* of the farthest and closest player with respect to the GC [25]. Together with the *distance* from their own GC, Sampaio et al. [38] suggested the *distance* between each player and the opponent´s GC in order to assess how player movement patterns are coordinated with all their teammates’ and opponents’ positioning expressed as a single value (i.e., GC).

Similarly to the player–space *distance* [5,21,22], the GC–space *distance* variable was suggested in order to assess the collective behaviour of particular sub-groups of players involved in the creation/prevention of goal scoring [39]. Specifically, Duarte et al. [39] implemented a 3 vs. 3 SSG task in which a line was drawn to simulate the task constraints of the 7-a-side offside rule for this age level (i.e., *defensive line*) and the *distance* measured between the GC and the defensive line in soccer. In the same line of research, Silva et al. [4] calculated the centroid’s *distance* to the goal centre defended by a goalkeeper and to the end line where the mini-goals were placed during several soccer SSGs. Thus, the GC–space *distance* has been suggested for assessing the relative position of the team, expressed as a single value, with respect to different types of goals.

### 4.4. Non-Linear Analysis Techniques

#### 4.4.1. Synchronisation

Regarding the relative phase for intra-team *distance* variables, Bourbousson et al. [16] suggested the analysis of the relationship between several playing positions (i.e., centre vs. guard; shooting guard vs. smart-forward; small forward vs. power forward). Further studies calculated the relative phase for all pairs of players [31] and suggested the assessment of the relative phase for every player with respect to the team and individual’s relative phase with the group measure [30]. On the other hand, regarding the relative phase for inter-team *distance* variables, Bourbousson et al. [16] assessed the five inter-team *distance* variables made between two players from each position, and due to the importance of the score, several studies suggested this analysis of the 1 vs. 1 *distance* close to the target (e.g., goal or basket) in order to assess the performance in these special play situations in team invasion sports [44,48].

Later, Travassos et al. [3] assessed the relative phase of five new types of *distance* variables: player–ball and player–teammate distances differentiating attacking and defending teams and the player–opponent distances. In addition, Travassos et al. [2] measured the relative phase for several new *distance* variables by the Hilbert transform: defending team–, attacking team–, teams–ball (i.e., GC–ball), and defending–attacking distances (i.e., GC–GC). These studies suggested that ball dynamics determine the relations between players [2,3]. In-phase attractions between players were reported to be stronger between defenders than attackers [3], and (a) stronger phase relations with the ball for the defending team than the attacking team [2] and (b) phase relations between each team and ball, and, to a lesser extent, between teams themselves, produced greater stability in the lateral (side-to-side) direction than the longitudinal (forward–backward) direction. The phase attraction between players and the ball showed how the mobile object and its dynamic is an important constraint on behaviour in a futsal game [3]. In addition, unlike other sports such as basketball [16], a greater in-phase relationship was found between defenders than attackers in the lateral directions [3]. In the same way, the second study found a higher in-phase relation between the defending team and the ball in both axes, and a higher in-phase relation between teams in the lateral direction [2].

The above-mentioned studies used the relative phase to measure synchronisation between two oscillators. In order to evaluate the synchronisation between more than two oscillators, Duarte et al. [30] applied the cluster method. Specifically, Duarte et al. [30] used the cluster method to measure player–team synchronisation (i.e., degree to which the behaviour of any one player in the team is synchronised to the movements of a team as a whole). In addition, after assessing the relative phase of all pairs of outfield players in several pre-season soccer matches, Folgado et al. [31] applied the k-means cluster analysis to capture intra-team *distance* variables with similar levels of synchronisation. This analysis was applied to the percentage of time of *distance* synchronisation and they classified each *distance* variable into one of three groups according to its synchronisation level: the higher, intermediate and lower synchronisation groups.

#### 4.4.2. Predictability

To our knowledge, Passos et al. [19] used the Approximate Entropy (ApEn) in team invasion sports for the first time. Specifically, the authors measured ApEn in the micro-structure 1 vs. 1, that is, in a player–opponent *distance* variable [19]. Based on the proposal of Stergiou et al. [49], the authors considered the number of observation windows to be compared (*m*) and the tolerance factor for which similarity between observation windows is accepted (*r*). Higher values of ApEn, close to 2, signified more complexity and less regularity and predictability. After analysing the relative position between defender and attacker during the micro-structure 1 vs. 1 near to the try line in rugby, they found that system complexity increased with changes in relations between players [19]. Moreover, the authors suggested the use of ApEn for other micro-structures involving more agents [19], and several studies have followed this proposal [20,50,51]. A further study suggested two normalised measures based on the original ApEn (i.e., normalised with respect to a maximum value of ApEn of a series of length N or of that particular set of points), which are less dependent on time series length, in order to measure the complexity and the regularity and predictability of a rugby union attacker–defender micro-structure [40].

Silva et al. [20] analysed the uncertainty of interpersonal *distance* values during soccer small-sided and conditioned games by means of sample entropy measures (SampEn), specifically the SampEn of *distance* to nearest opponent, i.e., the entropy of player–opponent distance. The use of SampEn instead of ApEn was suggested by Richman and Moorman [52] for two main reasons: a) ApEn was heavily dependent on the record length and is uniformly lower than expected for short records and b) it lacks relative consistency.

Also using SampEn, Barnabé et al. [32] measured the predictability of the team´s length (i.e., between the most forward and the most backward players) and width (i.e., between the farthest players on both sides) and stretch index. In addition, the same authors [32] used the cross-SampEn to assess the asynchrony of the same variables. Cross-SampEn was developed by Richman and Moorman [52] because Cross-ApEn presents the necessity for each template to generate a defined nonzero probability, and cross-SampEn remains relatively consistent for conditions where cross-ApEn does not. A new step was suggested by Gonsalves et al. [17], who, unlike Barnabé et al. [32], measured the predictability in the *distance* between all player distances formed by outfield players using ApEn.

## 5. Conclusions

Several types of *distance* variables have been suggested during the last two decades. According to the nature of the oscillators or points, they can be classified into player–player (i.e., player–opponent, player–teammate), player–space, player–ball, and GC–GC/player/space/goal *distance* variables. The measurement of the *distance* between players allows the assessment of the interaction between a couple of players, teammates or opponents. It is of special interest in those sports in which man-marking is commonly used, such as basketball and futsal, and in the micro-structure close to scoring situations in all team invasion sports. Moreover, player–player distances are used to measure the length and the width of the team and player–GC distances to assess the dispersion of the team. The player–space distances have been measured to assess the *distance* of a player (or team line) from relevant spaces, such as the target or the external borders of the playing space. Although these variables may be interesting considering each one independently, it would be worthwhile to analyse the relationship among them.

The application of the relative phase and entropy has allowed the analysis of the synchronisation and the complexity and regularity or predictability of several GC and distances tactical variables (i.e., *relative phase*, *cluster method*; *entropy*, ApEn, ApEn_ratioRandon_, ApEn_ratioSuffle_, SampEn, cross-SampEn, AMI). Usually, the relative phase has been used to measure the synchronisation between two oscillators (i.e., Hibert transform), but several authors have suggested the cluster method in order to evaluate the synchronisation among more than two oscillators. This suggestion comprises a more complex analysis of team invasion sports. Regarding entropy, different types of techniques have been suggested (i.e., ApEn, ApEn_ratioRandon_, ApEn_ratioSuffle_, SampEn, cross-SampEn). There is no consensus, and this makes the comparison among studies difficult.

## Figures and Tables

**Table 1:** Classification of the *distance* variables.

Variable	Group and Sub-Groups of Variables	Variables Included in Each Group
*Distance* variables	Distance between two points (i.e., GC of several players, players, space, ball)	
	*Player–player*	
	Player–opponent	Player–opponent. Team separateness
	Player –teammate	Player–teammate. Length; Width
	*Player–space*	Player–line. Player–goal.
	*Player–ball*	Player–ball
	*GC–GC*	GC–GC
	*GC–Player*	Own/opponent GC–player
	*GC–Space*	GC-defensive line /goal

GC: Geometrical centre. Italic for the main groups and no italic for the subgroups: *Player-player (main group)*; Player-opponent (subgroup); Player-teammate (subgroups); *Player-space (main group)*; *Player-ball (main group)*; *GC-GC (main group)*; *GC-player (main group)*; *GC-space (main group).*

**Table 2:** Origin and modifications of the *distance* variables.

Author	Type of *Distance* variable	Definition	Sport	Competition Level	Task	EPTS	Q
Passos et al. [24]	*Player–opponent*	Attacker–defender distance: interpersonal *distance* and relative velocity	Rugby	Young	1vs. 1 task	OPTs	87
Bourbousson et al. [16]		Player–opponent *distance* matched for playing position	Basketball	Professional	Match	OPTs	81
Silva et al. [20]		The TS for a team was defined as the sum of distances between each team player and the closest opponent.	Soccer	National-level and RLP-regional-level players	4 vs. (4+GK)	GPS	87
Silva et al. [36]		The average *distance* between all players and their closest opponent (TS)	Soccer	U-15	3 vs. 3 4 vs. 4 5 vs. 5	GPS	93
Silva et al. [4]		Teams’ horizontal and vertical opposing line-forces (i.e., the distances separating the teams’ vertical opposing line-forces and the distances separating the teams’ horizontal opposing line-forces)	Soccer	National-level and RLP-regional-level players	5 vs. 5 5 vs. 4 5 vs. 3	GPS	87
Shafizadeh et al. [26]		Closing *distance* gap between shooter and goalkeeper	Soccer	Professional	Match (1 vs. 1 direct shoot situations)	OPTs	93
Lames et al. [37]	*Player–teammate*	Range per team. Difference between max and min position of players except goalkeeper	Soccer	Professional	Match	OPTs	-
Bourbousson et al. [16]		The inter-team distances made between two players of each position	Basketball	Professional	Match	OPTs	81
Goncalves et al. [17]		Variability in the *distance* between players	Soccer	Professional	3 experimental conditions	GPS	87
Olthof et al. [18]		Represents the space between *goalkeeper* and nearest defender (defending line).	Soccer	Young	4 vs. (4 + GK)	LPS	93
Passos et al. [22]	*Player–space*	Player (attacker and defender)–try line distance	Rugby	Young	1 vs. 1	OPTs	93
Passos et al. [22]		Player (attacker and defender)–both lateral lines distance	Rugby	Young	1 vs. 1	OPTs	93
Vilar et al. [5]		Relative *distance* to the goal.	Futsal	Professional	Match (1 vs. 1 sequences)	OPTs	87
Esteves et al. [21]		The *distance* of the ball carrier to the basket at the time of either shooting or losing ball possession.	Basketball	Young	Match	OPTs	87
Yue et al. [7]	*Player–ball*	Player–ball *distance* in the x- and y-direction	Soccer	Professional	Match	OPTs	73
Frencken & Lemmink [6]	GC–GC	Distance between two GCs of the teams	Soccer	Elite Youth	4 vs. (4 + GK)	LPM	-
Frencken & Lemmink [6]	GC–Player	Distance between GCs and players	Soccer	Elite Youth	4 vs. (4 + GK)	LPM	-
Yue et al. [7]	*Own team GC–player*	*Averagedistance* between all players and the GC of the team [Radius]	Soccer		Match	OPTs	75
Bartlett et al. [23]		Radial, along pitch and across pitch Frobenius norm	Soccer	Professional	Match	OPTs	87
Sampaio & Maçãs [25]		*Absolutedistance* of each player from the GC of the team	Soccer	University Student	5 vs. 5	GPS	87
Sampaio & Maçãs [25]		Maximal *distance* of the farthest player from the GC of the team	Soccer	University Student	5 vs. 5	GPS	87
Sampaio & Maçãs [25]		Minimal *distance* of the nearest player from the GC of the team	Soccer	University Student	5 vs. 5	GPS	87
Sampaio et al. [38]	*Opponent team’s GC–player*	Distance between each player and the opponents’ centroid	Basketball	Junior	5 vs. 5	GPS	93
Sampaio et al. [38]	GC–Space	Distance between GCs and a point in the space	Basketball	Junior	5 vs. 5	GPS	93
Duarte et al. [39]	*GC–defensive line*	the smallest *distance* of the centroid to the defensive line using x-component motion values	Soccer	Young	3 vs. 3	OPTs	87
Silva et al. [4]	*GC–goal*	The centroid’s *distance* to the goal centre	Soccer	National-level and RLP-regional-level players	5 vs. 5 5 vs. 4 5 vs. 3	GPS	87

GC: geometrical centre; GK: Goalkeeper; GPS: Global Positioning Systems; LPM: local position measurement; LPS: local position system; OPTs: optic-based systems; Q: Quality score (%); TS: team separateness.

**Table 3:** Origin and modifications of the application of the data processing techniques in the *distance* variables.

Author	Variable	Sport	Competition Level	Task	EPTS	Q
*Relative phase*						
Passos et al. [19]	Player–opponent	Rugby	Young, national level	1 vs. 1	OPTs	81
Bourbousson et al. [16]	Player–teammate	Basketball	Professional	Match	OPTs	81
Bourbousson et al. [16]	Player–opponent	Basketball	Professional	Match	OPTs	81
Bourbousson et al. [15]	Stretch indexes	Basketball	Professional	Match	OPTs	81
Travassos et al. [3]	Player–ball for attacking and defending teams *	Futsal	National Futsal University	5 vs. (4 + GK)	OPTs	75
Travassos et al. [3]	Player–teammate for attacking and defending teams *	Futsal	National Futsal University	5 vs. (4 + GK)	OPTs	75
Travassos et al. [3]	Player–opponent *	Futsal	National Futsal University	5 vs. (4 + GK)	OPTs	75
Travassos et al. [2]	Defending team–ball	Futsal	National Futsal University	5 vs. (4 + GK)	OPTs	87
Travassos et al. [2]	Attacking team–ball	Futsal	National Futsal University	5 vs. (4 + GK)	OPTs	87
Travassos et al. [2]	Teams–ball	Futsal	National Futsal University	5 vs. (4 + GK)	OPTs	87
Duarte et al. [30]	Every player–team	Football	Professional	Match	OPTs	80
Duarte et al. [30]	Player–team *	Football	Professional	Match	OPTs	80
Folgado et al. [31]	Player–teammate *	Football	Professional	Match	GPS	87
*Entropy*						
Passos et al. [19]	Player–opponent	Rugby	Young, national level	1 vs. 1		81
Sampaio & Maçãs [25]	Absolute *distance* of each player from the GC of the team	Soccer	University Student	5 vs. 5	GPS	87
Sampaio & Maçãs [25]	Maximal *distance* of the farthest player from the GC of the team	Soccer	University Student	5 vs. 5	GPS	87
Sampaio & Maçãs [25]	Minimal *distance* of the nearest player from the GC of the team	Soccer	University Student	5 vs. 5	GPS	87
Fonseca et al. [40]	Player–opponent	Rugby	-	1 vs. 1		87
Silva et al. [20]	Player–opponent	Soccer	Young (regional and national level)	(4 + GK) vs. (4 + GK)	GPS	87
Barnabé et al. [32]	Player–teammate (team’ length)	Soccer	Young	(5 + GK) vs. (5 + GK)	GPS	80
Barnabé et al. [32]	Player–teammate (team width)	Soccer	Young	(5 + GK) vs. (5 + GK)	GPS	80
Barnabé et al. [32]	Player–GC (stretch index)	Soccer	Young	(5 + GK) vs. (5 + GK)	GPS	80
Goncalves et al. [17]	Player distances formed by the outfield teammates	Soccer	Professional	10 vs. 9 LSG	GPS	87

ApEn: Approximate entropy; GPS: Global Positioning System; LSG: large-sided game; OPTs: optic-based systems; game; Q: Quality score (%); SampEn: Sample entropy; *: cluster analysis was applied.
